# Plant Diversity Exerts a Stronger Influence than Short-Term Climate Manipulations on the Structure of Soil Bacterial Communities

**DOI:** 10.3390/microorganisms13122844

**Published:** 2025-12-15

**Authors:** Mingxuan Yi, Pengfei Cong, Dongming Zhang, Jiangong You, Yan Zhang, Wentao Jing, Liwen Shang

**Affiliations:** 1 Langfang Integrated Natural Resources Survey Center, China Geological Survey, Langfang 065000, China; yimingxuan@mail.cgs.gov.cn (M.Y.);; 2 Innovation Base for Natural Resources Monitoring Technology in the Lower Reaches of Yongding River, Geological Society of China, Langfang 065000, China; 3 Haihe River Basin North Natural Resources Field Scientific Observatory, Langfang Integrated Natural Resources Survey Center, China Geological Survey, Yongqing, Langfang 065699, China; 4 Innovation Base of Natural Resources Change Observation and Capital Monitoring in the Northern Haihe River Basin, China Society of Territorial Economists, Langfang 065000, China

**Keywords:** plant diversity, climate change, soil bacterial community, *Sphingomonas*, plant–soil feedback

## Abstract

Soil microbial communities face the combined pressures of climate change and biodiversity loss, yet how these stressors interact to shape ecosystem function remains a critical uncertainty. To investigate this, we established a constructed grassland plant community and conducted a fully factorial experiment manipulating plant diversity (1, 3, and 6 species), temperature (ambient, +2 °C), and precipitation (ambient, +50%). High-throughput 16S rRNA gene sequencing revealed that plant diversity exerted a stronger influence on soil bacterial community structure than did warming or precipitation changes. Beta diversity analysis revealed a distinct clustering of bacterial communities corresponding to the plant diversity gradient. This shift was characterized by a consistent enrichment of the metabolically versatile genus *Sphingomonas* in medium-diversity plots that experienced elevated precipitation, suggesting a predicted potential for enhanced organic matter decomposition. Despite overall stability in alpha diversity, the interaction between plant diversity and warming significantly modulated bacterial diversity and dominance patterns. Our findings highlight that plant diversity plays a key role in mediating soil bacterial responses to simulated climate factors in the short term. Incorporating these plant–soil feedback mechanisms into ecological models appears crucial for advancing predictions of ecosystem dynamics under future climate conditions.

## 1. Introduction

Global climate change is exerting unprecedented pressure on terrestrial ecosystems, notably through the dual stressors of plant diversity loss and alterations in climatic regimes, such as rising temperatures and shifting precipitation patterns [1,2,3]. Plant diversity plays a fundamental role in influencing the composition, structure, and functioning of soil microbial communities [4,5]. For instance, increased plant richness has been shown to enhance microbial diversity and reinforce associated ecological processes [6]. Within the complex interplay between vegetation and climate, shifts in plant species composition can modify the rhizosphere environment, thereby altering competitive and cooperative interactions among microorganisms [7]. These changes may favor microbial taxa adapted to the new conditions, ultimately reshaping the dominant structure of the soil microbial community [8]. A growing body of evidence suggests that plant communities can function as key biological buffers, mitigating climate-associated pressures on soil microbes by modulating the rhizosphere microenvironment [9,10,11,12]. Understanding this plant-mediated buffering capacity is essential to more accurately forecast the behavior and functionality of ecosystems under future climate scenarios.

Previous studies have shown that plant diversity serves as a primary driver of soil microbial community composition and function. This influence is largely mediated through two key mechanisms: diversification of root exudates and expansion of ecological niches [13,14]. For example, legumes such as *Medicago sativa* release flavonoids and other signaling compounds that attract and support rhizobia, enhancing nitrogen-fixing bacterial communities [15]. In contrast, grasses like *Lolium perenne* exude carbohydrates that promote decomposer bacteria and fungi [16]. This chemical diversity creates distinct microhabitats within the rhizosphere, selectively enriching taxa with complementary metabolic capabilities. Substantial evidence demonstrates positive correlations between plant species richness and microbial diversity [17,18], while plant genotypic variations significantly enhance fungal diversity [19,20,21]. Notably, these plant-driven effects on soil microbiota intensify over time [22]. In parallel, climate change factors exert direct influences on microbial assemblages through distinct pathways. Warming accelerates microbial metabolic rates and modifies competitive interactions, thereby driving community succession [23,24,25]. Altered precipitation regimes affect soil moisture dynamics, leading to redistribution of microbial resources [26,27,28]. These physiological adjustments ultimately manifest as modifications in functional gene composition [29,30,31]. The functional potential of the soil microbiome governs fundamental nutrient cycles. Moreover, it influences key ecosystem services, such as the remediation of contaminated soils. Recent advances highlight that microbial communities, particularly those enriched in diverse plant systems, can degrade agrochemical pollutants through specialized enzymatic mechanisms and collective behaviors mediated by quorum sensing [32,33]. This underscores that plant-driven shifts in microbial assembly could have profound implications for ecosystem resilience and functionality beyond carbon and nutrient cycling. Nevertheless, synthesizing predictions from studies that examine individual factors or paired combinations remains a considerable challenge. While recent research has made significant strides by exploring the interplay between plant diversity and specific climate factors, such as drought [34], as well as the combined effects of warming and altered precipitation [35]. To build upon this important work and capture the synergistic nature of global change drivers, our study employs a full-factorial experimental design that concurrently manipulates plant diversity (1, 3, and 6 species), temperature (ambient vs. +2 °C), and precipitation (ambient vs. +50% addition). This integrated experimental design allows us to disentangle the individual and interactive effects of these key drivers on soil microbial communities. The assembly of defined plant compositions establishes causal relationships between plant diversity and microbial responses, moving beyond correlative evidence. Through high-throughput sequencing of bacterial 16S rRNA genes, we examine multiple dimensions of microbial communities—including structure, diversity, and functional taxa—to uncover the mechanisms underlying their responses to changing aboveground conditions.

We propose the following hypotheses: (1) Plant diversity predominates over warming and precipitation changes in shaping soil bacterial community structure; (2) Higher plant diversity stabilizes microbial community structure and function under climate stress, maintaining alpha diversity despite environmental fluctuations; and (3) We further hypothesize that intermediate plant diversity, under specific temperature and moisture regimes, fosters distinct ecological niches. These niches select for bacterial taxa with specialized metabolic capabilities, such as organic matter decomposition. This multifactor experiment provides a mechanistic understanding of how plant diversity and climate drivers interact to regulate soil microbial communities. Our results offer critical insights for predicting initial ecosystem responses to environmental change and support biodiversity-based approaches to enhance ecological resilience in managed grasslands.

## 2. Materials and Methods

### 2.1. Study Area Description

**Figure 1:**
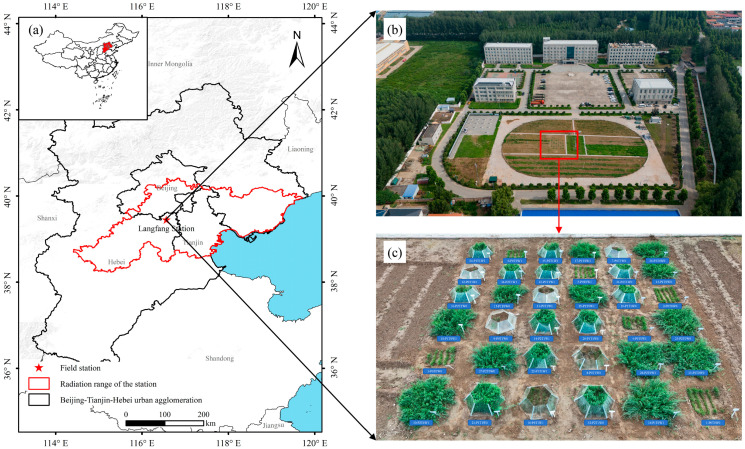
Overview of the study area. Note: (**a**) illustrates the geographical location, (**b**) shows an aerial view of the field station, and (**c**) details the layout of the simulated experimental plots.

This study was conducted at the simulated experimental field of the Northern Haihe River Basin Field Scientific Observation and Research Station for Natural Resource Elements (116.5601° E, 39.4512° N; Figure 1) (referred to as the “Langfang Station”). The station is located in Yongqing County, Langfang City, Hebei Province, within the northern part of the North China Plain and the middle-lower reaches of the Haihe River Basin. This area represents a critical transitional zone connecting the Beijing-Tianjin-Hebei urban agglomeration and its ecological hinterland. The terrain is flat and low-lying, with an average elevation of approximately 20 m above sea level. The region experiences a warm-temperate, semi-humid continental monsoon climate, characterized by a mean annual temperature of 11.5 °C and an average annual precipitation of approximately 550 mm (based on 1991–2020 data), with over 70% of rainfall concentrated between June and August. The annual potential evaporation is around 1800 mm. The soil at the experimental site is classified as loam.

### 2.2. Experimental Materials

Based on an original field survey of grassland communities in the Northern Haihe River Basin conducted by our team in 2024 (data not yet published elsewhere), the herbaceous plant communities in this basin are primarily composed of species from the Poaceae, Fabaceae, and Asteraceae families. Six common grassland species were selected, representing three key functional groups: the grasses *Leymus chinensis* and *Lolium perenne*; the nitrogen-fixing legumes *Medicago sativa* and *Melilotus officinalis*; and the forbs (Asteraceae) *Taraxacum mongolicum* and *Artemisia argyi*. All seeds were procured from Lüqin Seed Industry (Suqian City, China). The seed packages did not specify cultivar names; all seeds were common wild-type or standard commercial varieties suitable for regional grassland restoration. The inclusion of legumes was particularly important for their known role in nitrogen fixation, a key plant-driven process that influences soil microbiota [36].

### 2.3. Experimental Design

We employed a completely randomized block design with three factors, treating the block as a random effect. The experimental treatments consisted of: Plant diversity (P0: 1 species; P1: 3 species; P2: 6 species), Temperature (T0: ambient; T1: +2 °C), and Precipitation (W0: natural precipitation; W1: +50% precipitation). The +2 °C warming aligns with the upper limit of Paris Agreement targets, simulating a plausible future scenario. The 50% precipitation increase was chosen to represent a realistic extreme rainfall event in the region, based on historical climate data. These manipulations allow us to test grassland responses to projected climate conditions without inducing unrepresentative extreme stress in the short term. This resulted in 12 distinct treatment combinations (Table 1). Each combination was replicated three times, totaling 36 experimental plots (Figure 1c).

Sowing was carried out manually in early April 2025. Seeds were sown at a density targeting establishment of ~500 plants per m^2^. Following the design, grass species (*Leymus chinensis* and *Lolium perenne*) were drilled in rows 15 cm apart and covered with 1–2 cm of soil. In contrast, seeds of legumes (*Medicago sativa* and *Melilotus officinalis*) and Asteraceae species (*Taraxacum mongolicum* and *Artemisia argyi*) were mixed and broadcast evenly across the plot, then lightly raked into the soil (with Asteraceae seeds covered by ≤1 cm of soil). Standard field management practices, including irrigation, fertilization, weed control, and pest management, were consistently applied to ensure optimal plant growth.

Treatment Duration and Sampling: The climate manipulations (warming and increased precipitation) commenced on 1 May 2025. The treatments were maintained for a total of three months, concluding with the final soil sampling on 1 August 2025. Temperature elevation was achieved using open-top chambers (OTCs). These hexagonal chambers were constructed from transparent polyvinyl chloride panels with a light transmittance exceeding 90%. The OTCs measured 1.2 m in diameter at the base, 0.7 m at the top, and 0.4 m in height. Monitoring of temperature inside the OTCs throughout the experimental period confirmed that the warming treatment successfully increased the air temperature by an average of 1.5–2.5 °C compared to the ambient control, which is consistent with the target of +2 °C. Supplemental precipitation was simulated manually using watering cans. Based on real-time rainfall data from a miniature weather station within the experimental field, supplementary irrigation was applied on the second and fourth days following a natural rainfall event. Each supplemental event delivered a water volume equivalent to 50% of the recorded natural rainfall.

### 2.4. Sample Collection

Samples were collected on 1 August 2025. From the center of each plot, we collected soil samples from the 0–20 cm depth using a 5 cm diameter corer. This depth was selected as it encompasses the primary root zone and the most biologically active layer of the soil profile, where the majority of plant-microbe interactions occur. The collected soil from each plot was homogenized and sequentially passed through 80-mesh and 100-mesh sieves. Approximately 100 g of the sieved soil were then placed into a sterile bag. All samples were preserved on dry ice and transported immediately to the laboratory. Each sample was divided into two aliquots: one was stored at −80 °C for subsequent DNA extraction and high-throughput sequencing, while the other was air-dried for physicochemical analysis.

### 2.5. Sample Analysis

#### 2.5.1. DNA Extraction

Total microbial genomic DNA was extracted from soil samples following the manufacturer’s protocol for the E.Z.N.A.^®^ soil DNA kit (Omega Bio-tek, Norcross, GA, USA). The quality of the extracted DNA was assessed via 1% agarose gel electrophoresis, and its concentration and purity were determined using a NanoDrop2000 spectrophotometer (Thermo Scientific, Waltham, MA, USA).

#### 2.5.2. PCR Amplification and Library Preparation

The extracted DNA served as the template for PCR amplification of the V3-V4 hypervariable region of the bacterial 16S rRNA gene, using barcoded primers 338F and 806R. The PCR reaction mixture (20 μL total volume) contained: 4 μL of 5× TransStart FastPfu Buffer, 2 μL of 2.5 mM dNTPs, 0.8 μL each of the forward and reverse primers (5 μM), 0.4 μL of TransStart FastPfu DNA Polymerase, and 10 ng of template DNA. The amplification protocol was as follows: initial denaturation at 95 °C for 3 min; 27 cycles of denaturation at 95 °C for 30 s, annealing at 55 °C for 30 s, and extension at 72 °C for 30 s; followed by a final extension at 72 °C for 10 min. The resulting PCR products were purified by extraction from a 2% agarose gel and quantified using Qubit 4.0. Sequencing libraries were constructed using the NEXTFLEX Rapid DNA-Seq Kit (Bioo Scientific Corporation, Austin, TX, USA) and sequenced on the Illumina Nextseq2000 platform. Sequencing generated an average of 25,088 high-quality reads per sample, targeting an amplicon length of ~460 bp for the bacterial 16S rRNA V3-V4 region [37].

#### 2.5.3. Bioinformatics Analysis

Raw paired-end sequences were processed for quality control and assembly using fastp (v0.19.6) and FLASH (v1.2.11), respectively. During quality control, reads were trimmed by removing low-quality bases (average quality score < 20) and truncated at the first instance of a base with quality score < 15. Reads shorter than 50 bp after trimming were discarded. Denoising and Amplicon Sequence Variant (ASV) generation were performed using the DADA2 plugin within the QIIME2 pipeline (version 2023.9). ASVs are high-resolution operational taxonomic units that represent exact biological sequences, providing finer taxonomic resolution than traditional OTU clustering methods. Taxonomic assignment of ASVs was carried out against the SILVA 138 SSU rRNA database. To account for variations in sequencing depth, all samples were rarefied to 25,088 sequences per sample, a standard normalization procedure that mitigates biases arising from unequal sequencing efforts across samples, achieving a Good’s coverage of 99.09%. A Venn diagram was constructed based on observed ASVs to visualize the number of unique and shared bacterial taxa across treatment groups.

### 2.6. Statistical Analysis

All statistical analyses of soil microorganisms were performed on the Majorbio Cloud Platform (https://cloud.majorbio.com accessed on 20 September 2025). Alpha diversity indices (Chao1, Shannon and Simpson) were calculated using mothur (version 1.30) on the rarefied ASV table. Differences between groups were assessed using the non-parametric Kruskal–Wallis H test, followed by pairwise Wilcoxon rank-sum test for post hoc comparisons where applicable [38]. To identify microbial taxa differentially enriched among treatments, Linear Discriminant Analysis Effect Size (LEfSe) analysis was performed using an LDA score threshold of >2.0 and a significance alpha of 0.05. Principal Coordinates Analysis (PCoA) based on Bray–Curtis distances was performed to visualize community differences, and the significance of these differences was tested using Permutational Multivariate Analysis of Variance (PERMANOVA) and ANOSIM. Statistical analysis of variance (ANOVA) was performed using SPSS software (version 23.0).

## 3. Results

### 3.1. Interactive Effects of Plant Diversity and Climate Factors on Soil Physicochemical Properties

Analysis of variance indicated no significant differences in soil temperature, moisture, or electrical conductivity across the different treatment combinations (*p* > 0.05; Table 2). Soil temperature ranged between 30.87 and 34.97 °C, moisture content varied from 22.0% to 27.3%, and electrical conductivity values fell within 187 to 216.67 μS/cm.

The absence of significant variation in these fundamental soil physical parameters is a critical and insightful finding. Although the OTC warming system and supplemental watering directly targeted the near-surface microclimate, they did not induce lasting, significant alterations in the measured soil temperature, moisture, or salinity at the root zone level within the timeframe and intensity of our experiment. This strongly suggests that the plant community itself, likely through plant–soil feedback mechanisms, can be a major factor influencing belowground processes under these specific conditions. 

### 3.2. Soil Bacterial OTU Clustering Under Plant Diversity and Climate Treatments

**Figure 2:**
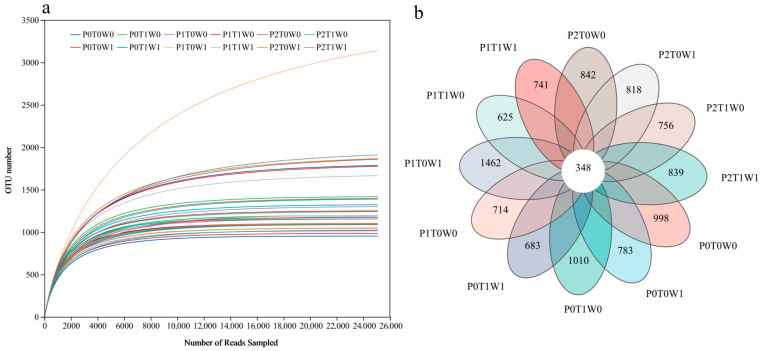
Soil sample sequencing data. (**a**) Bacterial community rarefaction curves. (**b**) Venn diagram of soil microbial OTUs across different treatments. Different colors represent different treatments. Overlapping areas indicate OTUs shared among multiple treatments, while non-overlapping sections represent OTUs unique to a specific treatment. Numbers correspond to the count of OTUs.

Sequencing results demonstrated adequate depth, as shown by the saturation of bacterial community dilution curves (Figure 2a) and a Goods coverage estimate of 0.99, confirming that the sequencing reliably captured the soil microbial community structure. To decipher the similarities and differences in microbial community composition across treatments, a Venn diagram was constructed based on observed species (Figure 2b). A total of 15,704 bacterial OTUs were detected. Strikingly, only 348 OTUs (2.21% of the total) were shared across all treatments. The P1T0W1 treatment (medium plant diversity, no warming, increased precipitation) harbored the highest number of unique OTUs (1462, accounting for 9.31% of the total), whereas the P0T1W1 treatment (single plant species, warming, increased precipitation) contained the fewest (683 OTUs, 4.35% of the total). Together, this delineates a limited core microbiome shared across all conditions and identifies a treatment-specific assemblage. This pattern indicates that intermediate plant diversity, combined with ample moisture but without warming, created the most distinct ecological niche space, supporting a more unique set of bacterial taxa compared to species-poor or stressed systems.

### 3.3. Effects of Plant Diversity and Climate Treatments on Soil Microbial Diversity

#### 3.3.1. Alpha Diversity of Soil Microbiota Across Treatment Combinations

We assessed the alpha diversity of soil bacterial communities under different treatments using the Chao1 richness index, Shannon diversity index, and Simpson dominance index. Although no statistically significant differences (*p* > 0.05) were detected among treatment groups, considerable variation in the mean values of the Chao1 index was observed numerically.

Community richness, as indicated by the Chao1 index (Table 3), ranged from an average of 1044.77 to 1930.22 across treatments. The highest richness was recorded in the P1T0W1 treatment (medium plant diversity, no warming, increased precipitation), with a mean of 1930.22, whereas the P1T1W0 treatment (medium plant diversity, warming, ambient precipitation) showed the lowest mean value of 1044.77. Although the richness in P1T0W1 was approximately 1.85 times that of P1T1W0, this difference was not statistically significant, which may be attributed to high within-group variation (e.g., standard error of 790.26 in P1T0W1).

In terms of community diversity and dominance, all treatments exhibited high homogeneity. The Shannon index values were tightly clustered between 6.35 and 6.60, indicating highly stable species diversity across treatments. Similarly, Simpson index values ranged from 0.0029 to 0.0064, consistently approaching zero, which reflects high species evenness and the absence of a single dominant taxon in all soil bacterial communities. Statistical analysis confirmed no significant differences between any two treatment groups for either the Shannon or Simpson index (*p* > 0.05).

In summary, although variations in plant diversity, warming, and precipitation led to considerable fluctuations in bacterial richness (Chao1 index), they did not significantly alter overall community diversity (Shannon index) or evenness (Simpson index). These findings suggest that the soil microbial community maintained a consistent level of diversity and evenness under the current experimental intensity of environmental perturbations, despite numerical variations in richness.

#### 3.3.2. Beta Diversity of Soil Microbiota Across Treatment Combinations

**Figure 3:**
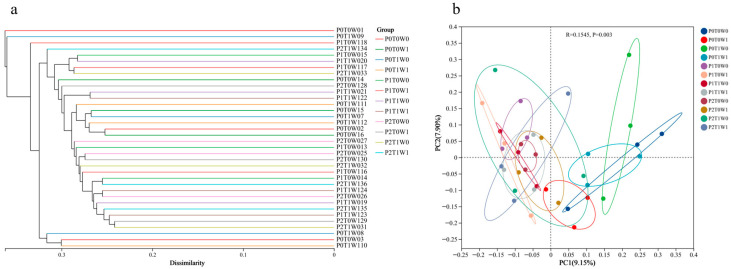
Beta diversity analysis of different soil samples. (**a**) Clustering analysis; (**b**) Principal coordinate analysis (PCoA).

Beta diversity analysis further elucidated the structural differences in bacterial communities among treatments. Clustering analysis (Figure 3a) showed that replicate samples from the same treatment (e.g., P0T0W0, P2T1W1) first clustered into minor branches at low dissimilarity levels (<0.1). These subsequently aggregated into larger branches corresponding to plant diversity levels (P0 → P1 → P2), forming a clear gradient from single-species to medium-diversity and high-diversity communities at dissimilarity thresholds between 0.1 and 0.3. In contrast, the effects of climate factors were not distinctly evident. Principal coordinate analysis (PCoA, Figure 3b) further confirmed clear separation in bacterial community structure among the different treatments. These results demonstrate that plant diversity is the dominant factor regulating community structure differentiation. The low-diversity systems (P0), likely due to missing functional groups, compressed ecological niches, and limited resource-use strategies, exhibited microbial community structures that significantly diverged from medium- and high-diversity systems.

### 3.4. Analysis of Soil Microbial Community Composition Under Plant Diversity and Climate Treatments

#### 3.4.1. Analysis of Soil Bacterial Community Structure at the Phylum Level

**Figure 4:**
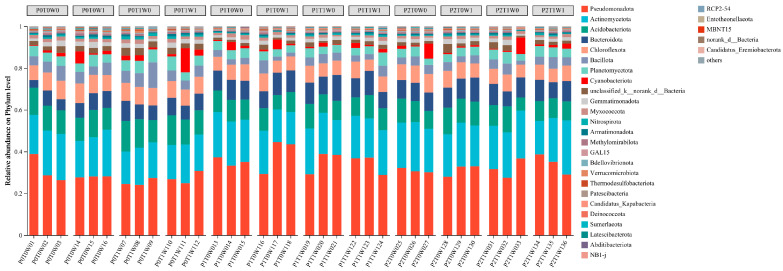
Soil bacterial community composition at the phylum level.

The overall structure of the soil bacterial community differed significantly among the different treatment combinations, as indicated by ANOSIM analysis (R = 0.1545, *p* = 0.002) and supported by the NMDS stress value (0.142, <0.2). Further analysis using LEfSe identified specific bacterial taxa that were differentially abundant in response to the treatments. At the phylum level (Figure 4), Pseudomonadota, Actinomycetota, Acidobacteriota, Bacteroidota, Chloroflexota, and Bacillota as the dominant phyla (relative abundance > 5%). Pseudomonadota was consistently the most abundant phylum across all treatments, though its relative abundance varied substantially, ranging from 24.08% to 44.58%. Actinomycetota (15.44–25.89%) and Acidobacteriota (6.29–14.57%) maintained stable and significant proportions. Notably, Cyanobacteriota remained below 2% in most samples but exhibited a marked increase (11.57%) in one replicate of the P0T1W1 treatment, indicating a potential bloom under specific conditions.

#### 3.4.2. Analysis of Soil Bacterial Communities at the Genus Level

**Figure 5:**
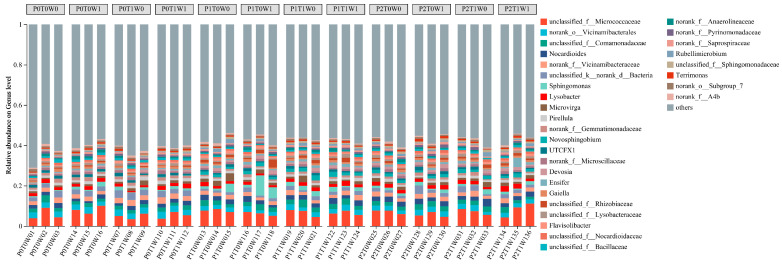
Soil bacterial community composition at the genus level.

At the genus level, the dominant bacterial taxa varied considerably among treatments (Figure 5). *Nocardioides* and *Lysobacter* were ubiquitously present as core genera, with relative abundances exceeding 1% in multiple treatments. In low-diversity plots (P0, single species), *Nocardioides* and *Lysobacter* dominated, with *Nocardioides* reaching 2.00% in both P0T0W0 and P0T1W1 treatments. A distinct community shift occurred under medium plant diversity (P1, three species), where *Sphingomonas* emerged as the predominant genus. Its abundance peaked at 5.61% in the P1T0W1 treatment (medium diversity, increased precipitation). This suggests that the specific root exudate profile and microenvironment generated by this particular plant diversity level, especially under increased moisture, acted as a strong biotic filter, selectively enriching for metabolically versatile taxa like *Sphingomonas*. *Microvirga* and *Novosphingobium* also became consistently prominent members within this group. In high-diversity plots (P2, six species), the community was characterized by a core of *Nocardioides* and *Lysobacter*, but with notably elevated abundances of *Ensifer*, *Novosphingobium*, and *Microvirga*.

## 4. Discussion

### 4.1. The Dominant Role of Plant Diversity in Shaping Soil Bacterial Communities

Our findings establish plant species richness as a key driver of soil bacterial community structure, with effects that were more pronounced than those of our experimental warming and altered precipitation treatments. The minimal direct effects of the climate manipulations are likely attributable to their relatively low intensity and the short-term nature of our experiment. Beta diversity analysis revealed a consistent and sequential shift in microbial composition along the plant diversity gradient, whereas the effects of temperature and precipitation treatments were comparatively minor. This finding contrasts with the conventional view that microbial communities in grassland ecosystems are primarily governed by the direct effects of climate [39,40,41,42,43]. By coupling three key drivers in our experimental design, we demonstrate that on short temporal scales and with moderate-intensity climate manipulations, the biotic filtering imposed by the plant community acts as a particularly strong determinant of bacterial assembly in our system. This conclusion aligns with the reports by Zhou et al. [44], Wang et al. [45], and Yang et al. [46]. Furthermore, while existing literature suggests that the influence of plant diversity on soil microbiota intensifies over time [22], our work provides new evidence that this plant-driven dominance persists even under concurrent short-term climate stressors. Consequently, we posit that within the intertwined context of biodiversity loss and climate change, the degradation of plant diversity may exert a more substantial impact on soil microbial communities than the direct ramifications of shifts in temperature and moisture.

### 4.2. Potential Mechanisms Underlying Plant Diversity Effects

The dominant role of plant diversity in shaping soil bacterial community assembly can be attributed to its direct modification of the rhizosphere environment through biological mechanisms [47,48]. Diverse plant communities release a variety of root exudates, including chemical signals and nutritional substrates, which create a heterogeneous chemical landscape in the rhizosphere. These exudates function as selective filters that enrich specific bacterial taxa possessing corresponding metabolic capabilities, thereby directly steering community composition [49,50]. Furthermore, increased plant richness enhances niche differentiation through divergent root architectures and litter inputs. The resulting physical and biological complexity supports a broader range of habitats for microorganisms, which helps sustain higher functional diversity and reduces competitive exclusion [51,52]. Thus, the coexistence of multiple plant species fosters a highly heterogeneous soil system capable of maintaining more diverse microbial lineages.

Critically, our study observed no significant changes in the soil parameters we measured (temperature, moisture, and electrical conductivity) following warming and precipitation treatments (Table 2). This strongly implies that the plant community stabilized the micro-environment—likely through canopy shading, transpiration, and soil structure improvement [53]. Such a buffering effect likely mitigated the direct physical impacts of climatic stressors, thereby allowing the signal of plant diversity to emerge as the dominant force in community assembly [54,55]. Therefore, plant diversity not only exerts strong direct effects but also modulates external stress by establishing a biological buffer, ultimately governing the community assembly process.

### 4.3. Microbial Community Stability and Structural Reorganization

Our analysis revealed consistent alpha diversity indices across all treatments, with no significant effects of climate manipulations on species richness or evenness. This pattern indicates considerable functional redundancy within the microbial communities. The observed stability aligns with the minimal diversity shifts reported by Wang et al. (2025) [24] under warming-only conditions. However, our multi-factor experiment extends this understanding by demonstrating that such resilience becomes particularly pronounced in diverse plant systems. Concurrently, we detected significant divergence in soil microbial beta diversity along the plant diversity gradient. This finding reveals a substantial restructuring of community composition, even as taxonomic richness remained stable, reflecting systematic shifts in the relative abundance of bacterial taxa in response to the plant diversity gradient [56,57]. The preservation of alpha diversity is consistent with the concept of a maintained functional potential, whereas the shifts in beta diversity indicate an adaptive reorganization to new ecological conditions [58]. In our study, plant diversity emerged as the critical driver of this microbial restructuring process. This interpretation is reinforced by our observation of a very limited core microbiome (only 2.21% of OTUs shared across all treatments). This small, stable core likely underpins basic functional stability, while the majority of compositional shifts occurred among the more dynamic, plant diversity-filtered taxa. It systematically reshapes species composition while maintaining the overall community diversity. We propose that this dual pattern of “stability and remodeling” likely enhances the stability of ecosystem functioning in response to environmental changes [59].

### 4.4. Response of Key Bacterial Taxa and Ecological Implications

The specific responses of bacterial taxa provide critical insights into the functional consequences of plant diversity gradients. Our study identified a significant enrichment of *Sphingomonas* under medium diversity with increased precipitation. This pattern represents a direct outcome of the interaction between specific plant community composition and water availability [60]. In soil ecosystems, *Sphingomonas* are recognized as metabolically versatile generalists, harboring enzymatic systems capable of degrading diverse complex organic compounds, including recalcitrant aromatics [61,62]. Beyond nutrient cycling, this metabolic versatility is directly relevant to the microbial remediation of soil contaminants. Their extensive biodegradative enzymatic machinery, coupled with community-level coordination mechanisms such as quorum sensing, positions them as key agents in mitigating the adverse impacts of agrochemicals on soil health and microbial diversity [63,64,65].

Beyond *Sphingomonas*, distinct bacterial groups were enriched across the plant diversity gradient. Low-diversity treatments were consistently dominated by generalist or stress-tolerant taxa such as Nocardioides and Lysobacter. In contrast, high-diversity treatments supported a more complex assemblage with prominent populations of Ensifer, Novosphingobium, and Microvirga (Figure 5). The selective enrichment of *Sphingomonas* under these conditions suggests that medium-diversity plant communities, when combined with elevated soil moisture, establish a distinct biochemical niche [66] that not only promotes nutrient turnover but may also enhance the ecosystem’s intrinsic potential for pollutant degradation. Consequently, the observed enrichment of *Sphingomonas* implies a potential connection between plant diversity gradients and key soil processes; however, direct functional evidence remains essential to confirm the implications for nutrient cycling and remediation potential [59,67,68].

### 4.5. Conceptual Integration and Perspectives

**Figure 6:**
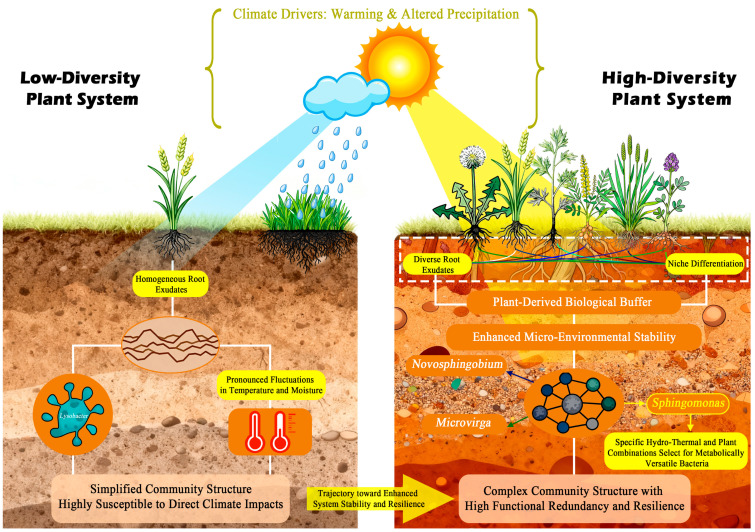
Schematic illustration of the proposed mechanisms whereby plant diversity serves as the primary driver of soil bacterial community structure and function under climate change.

Based on our findings, we propose a conceptual model elucidating the mechanisms through which plant diversity governs soil bacterial community structure and function within plant–soil-climate feedback loops (Figure 6). The model posits that in species-poor plant communities, limited root exudate diversity and reduced structural complexity collectively generate a homogenized rhizosphere environment. This results in a less diverse soil bacterial community dominated by a few stress-tolerant or generalist taxa, such as Nocardioides and Lysobacter observed in our low-diversity treatments. In contrast, species-rich plant communities foster a complex and stabilized rhizosphere environment through diversified root exudates and increased niche heterogeneity. This robust plant-derived buffer effectively mitigates the direct impacts of climatic stressors and stabilizes the soil micro-environment. Within this stable and resource-rich setting, a greater array of competitive and facilitative interactions among bacterial taxa can develop. This not only supports higher overall microbial diversity and complexity but also promotes the emergence of specific functional groups. The marked enrichment of *Sphingomonas* under medium diversity with increased precipitation (P1W1) exemplifies this mechanism, wherein specific plant diversity interacts with climatic factors to create a distinct niche favoring taxa with specialized metabolic capabilities.

This perspective carries significant practical implications for ecosystem management under climate change. Our findings provide robust scientific support for biodiversity-based adaptation strategies. Specifically, for grassland management, our results advocate for the conservation and restoration of high-diversity plant communities as a viable strategy to enhance soil health and ecosystem stability against climatic perturbations. By fostering complex microbial networks with greater functional redundancy and stability, diverse grassland ecosystems may exhibit improved adaptability to climatic perturbations, thereby maintaining critical processes such as nutrient cycling and organic matter decomposition.

While this study offers novel insights, certain limitations should be acknowledged. The relatively short experimental duration constrains the extrapolation of our findings to long-term climate change scenarios, where microbial community dynamics may differ. Furthermore, our analysis of bacterial community structure based on 16S rRNA gene sequencing does not directly reveal functional dynamics. Future research should therefore investigate whether these plant diversity buffering effects persist over longer timescales. A critical next step is to examine how climate change factors (warming and altered precipitation) modify the composition and quantity of root exudates from different plant families, and how these changes subsequently reshape microbiome assembly and function. Such efforts should integrate metagenomic and metabolomic analyses to directly link shifts in plant diversity to microbial community structure and functional activity, thereby providing a mechanistic understanding of Plant–Soil feedback under climate change.

## 5. Conclusions

In conclusion, our short-term experiment demonstrates that plant diversity, rather than moderate climate manipulations, is the principal driver of soil bacterial community structure in our grassland system. This was evidenced by the distinct clustering of microbial communities along the plant richness gradient. While the communities underwent significant structural reorganization, they maintained stable alpha diversity, suggesting a reshuffling of taxa rather than a net loss.

The observed patterns are likely governed by plant-mediated biotic filtering. Diverse root systems create heterogeneous rhizosphere environments that selectively enrich for specific bacterial taxa. This mechanism is exemplified by the notable increase in the metabolically versatile genus *Sphingomonas* under specific combinations of plant diversity and soil moisture, indicating the formation of distinct, plant-driven ecological niches. However, as our study relies on 16S rRNA data, the explicit functional roles of these key taxa and their direct relevance to processes like microbial remediation remain inferred and require validation through functional genomics.

Our findings highlight the practical importance of preserving and restoring plant diversity as a key management strategy. This approach is central to enhancing grassland ecosystem resilience, as it stabilizes the soil microenvironment and buffers microbial communities against climatic stressors. Future research should prioritize long-term studies that integrate metagenomics and metabolomics to directly quantify the functional linkages between plant diversity, microbial assembly, and ecosystem processes under sustained environmental change.

## Figures and Tables

**Table 1:** Different treatment combinations.

Treatment	Plant Diversity	Temperature	Precipitation
P0T0W0	1 species (monoculture)	No warming (control)	Natural precipitation (control)
P0T0W1	1 species (monoculture)	No warming (control)	Increased precipitation (+50%)
P0T1W0	1 species (monoculture)	Warming (+2 °C)	Natural precipitation (control)
P0T1W1	1 species (monoculture)	Warming (+2 °C)	Increased precipitation (+50%)
P1T0W0	3 species (medium)	No warming (control)	Natural precipitation (control)
P1T0W1	3 species (medium)	No warming (control)	Increased precipitation (+50%)
P1T1W0	3 species (medium)	Warming (+2 °C)	Natural precipitation (control)
P1T1W1	3 species (medium)	Warming (+2 °C)	Increased precipitation (+50%)
P2T0W0	6 species (high)	No warming (control)	Natural precipitation (control)
P2T0W1	6 species (high)	No warming (control)	Increased precipitation (+50%)
P2T1W0	6 species (high)	Warming (+2 °C)	Natural precipitation (control)
P2T1W1	6 species (high)	Warming (+2 °C)	Increased precipitation (+50%)

Note: The table presents a factorial experimental design combining three factors: plant diversity (P), temperature (T), and precipitation (W).

**Table 2:** Variations in soil temperature, moisture, and electrical conductivity under different treatments.

Treatment	Temperature (°C)	Moisture (%)
P0T0W0	34.97 ± 2.63 a	23.63 ± 2.54 a
P0T0W1	31.07 ± 0.98 a	27.3 ± 1.59 a
P0T1W0	31.97 ± 1.24 a	24.9 ± 1.82 a
P0T1W1	32.7 ± 2.26 a	25.53 ± 0.87 a
P1T0W0	31.57 ± 0.88 a	22.4 ± 1.45 a
P1T0W1	33.8 ± 2.84 a	22.53 ± 1.66 a
P1T1W0	33.23 ± 1.6 a	23.93 ± 1.35 a
P1T1W1	31.37 ± 1.32 a	23.67 ± 1.47 a
P2T0W0	32.3 ± 1.43 a	23.33 ± 1.1 a
P2T0W1	33.13 ± 1.22 a	22 ± 1.62 a
P2T1W0	33.67 ± 2.55 a	21.17 ± 1.7 a
P2T1W1	30.87 ± 0.87 a	26.2 ± 0.53 a

Note: Data are presented as mean ± standard error with 3 replicates per treatment. Different lowercase letters within a column indicate significant differences among treatments (*p* < 0.05).

**Table 3:** Microbial alpha diversity indices in soil samples.

Treatment	Chao1 Richness	Shannon Diversity	Simpson Dominance
P0T0W0	1288.15 ± 264.62 a	6.42 ± 0.18 a	0.0063 ± 0.00154 a
P0T0W1	1413.94 ± 202.08 a	6.53 ± 0.1 a	0.0061 ± 0.00099 a
P0T1W0	1259.2 ± 81.4 a	6.6 ± 0.05 a	0.0029 ± 0.00028 b
P0T1W1	1195.93 ± 69.41 a	6.49 ± 0.1 a	0.004 ± 0.00091 ab
P1T0W0	1114.25 ± 50.53 a	6.36 ± 0.04 a	0.0056 ± 0.00093 ab
P1T0W1	1930.22 ± 790.26 a	6.52 ± 0.22 a	0.0056 ± 0.00033 ab
P1T1W0	1044.77 ± 34.73 a	6.35 ± 0.05 a	0.0048 ± 0.00087 ab
P1T1W1	1327.72 ± 178.56 a	6.53 ± 0.13 a	0.0045 ± 0.00065 ab
P2T0W0	1465.04 ± 236.53 a	6.57 ± 0.12 a	0.0052 ± 0.00089 ab
P2T0W1	1361.09 ± 290.4 a	6.57 ± 0.13 a	0.0036 ± 0.00037 ab
P2T1W0	1221.96 ± 107.96 a	6.42 ± 0.09 a	0.0059 ± 0.00076 ab
P2T1W1	1454.97 ± 266.45 a	6.47 ± 0.06 a	0.0064 ± 0.00153 a
P	NS	NS	NS
T	NS	NS	NS
W	*	NS	NS
P × T	NS	*	*
P × W	NS	NS	NS
T × W	NS	NS	NS
P × T × W	NS	NS	NS

Note: Data are presented as mean ± standard error. Different lowercase letters within a column indicate significant differences (*p* < 0.05), while the same letters indicate no significant difference (*p* > 0.05). * *p* < 0.05, NS not significant.

## Data Availability

The original contributions presented in this study are included in the article. Further inquiries can be directed to the corresponding author.
